# Diet Impact on the Development and Survival of *Oiceoptoma thoracicum* (Coleoptera: Silphidae)

**DOI:** 10.1093/jme/tjac129

**Published:** 2022-09-08

**Authors:** Jarin Qubaiová, Pavel Jakubec, Santiago Montoya-Molina, Martin Novák, Hana Šuláková

**Affiliations:** Department of Ecology, Faculty of Environmental Sciences, Czech University of Life Sciences Prague, Kamýcká 129, CZ-165 00 Praha, Suchdol, Czech Republic; Department of Ecology, Faculty of Environmental Sciences, Czech University of Life Sciences Prague, Kamýcká 129, CZ-165 00 Praha, Suchdol, Czech Republic; Department of Ecology, Faculty of Environmental Sciences, Czech University of Life Sciences Prague, Kamýcká 129, CZ-165 00 Praha, Suchdol, Czech Republic; Department of Ecology, Faculty of Environmental Sciences, Czech University of Life Sciences Prague, Kamýcká 129, CZ-165 00 Praha, Suchdol, Czech Republic; Department of Ecology, Faculty of Environmental Sciences, Czech University of Life Sciences Prague, Kamýcká 129, CZ-165 00 Praha, Suchdol, Czech Republic; Police of the Czech Republic, Institute of Criminalistics Prague, P.O. Box 62/KUP, CZ-170 89 Praha, Czech Republic

**Keywords:** Carrion beetle, development time, mortality, nutritional ecology, larval instar

## Abstract

We assessed the influence of diet on the development and survival in the immature stages of the necrophagous beetle *Oiceoptoma thoracicum* (Linnaeus, 1758). The species is frequently observed on large cadavers, including humans, and thus can be of potential forensic relevance. We compared multiple meat tissues from three animal sources, and detected the optimal diet for rearing the species for further entomological and forensic objectives. We reared 203 individuals to adulthood at the constant temperature of 20°C. Our results represent the first robust data set of the development time for this species. They further confirmed a significant relationship between survival and the type of diet, as the highest survival rates were detected in larvae fed with both pork liver and pork muscle.

Diet plays an essential role in the general performance of insects, as it can significantly affect their biology (e.g., size, development, and growth rates), as well as their survival and reproduction (House 1969, 1974; Scriber and Slansky 1981, Nation 2015). This effect should not be ignored especially when rearing the larval stages of forensically relevant species. As discrepancies in the rearing methodologies, using diets from different animal species and tissues, can lead to failures in breeding these species and, more importantly, to significantly different developmental models (Byrd and Tomberlin 2019, Qubaiová et al. 2021).

Elucidating the larval nutritional requirements, which often differ from those of the adults, and determining the optimal food sources, is critical for attaining reliable reference developmental data. These in turn, provide more precise estimations of the minimum post-mortem interval (PMImin) during the criminal and legal investigations (Wells and LaMotte 2009, Tarone and Benoit 2019).

Most diet studies involving forensically important insects were performed on Diptera (e.g., Thyssen et al. 2014, Bernhardt et al. 2017, Bambaradeniya et al. 2019, Rogers et al. 2021), yet current areas of research gradually include necrophagous beetles as well (e.g., Frątczak-Łagiewska et al. 2020, Jakubec et al. 2021, Qubaiová et al. 2021). The primary interest resides in their longer life cycle, which can sequentially extend the PMI estimation period. Furthermore, beetle larvae can be reared individually or in very small groups, which has diagnostic benefits (Haskell et al. 1997, Ridgeway et al. 2014).

Genus *Oiceoptoma* Leach, 1815 is considered of forensic relevance, since it frequently appears on carcasses associated with crime scenes (Byrd and Castner 2009). The genus consists of nine described species (Newton 2020) of which most inhabit Asia, and three are found in North America. We focused this study on *Oiceoptoma thoracicum* (Linnaeus, 1758), the only species that extends to Europe (Ratcliffe 1996) and is distributed throughout the Palearctic realm (Růžička et al. 2004, Newton 2020). The sp. *O. thoracicum* inhabits and reproduces mainly in forest biotopes (Kočárek 2001, 2003; Dekeirsschieter et al. 2011a), seldom in open habitats (Růžička 1994, Jakubec and Růžička 2015). Therefore, it is suggested that the species could be a valuable indicator in forensic investigations, particularly for the post-mortem body relocation cases (Matuszewski et al. 2010, 2013; Charabidze et al. 2017). In the Czech Republic, the species was observed on 4.56% of deceased human bodies in 395 forensic cases between 2002 and 2021 (Šuláková, unpublished data).

The ecology of *O. thoracicum* is relatively unknown and is mainly based on anecdotal observations. In Europe, it was revealed that the species has two activity peaks during spring and summer (Kočárek and Benko 1997, Esh and Oxbrough 2021). Generally, the adults feed on diverse diets, namely decaying carrion, dung, and mushrooms (Dekeirsschieter et al. 2011b). Nonetheless, the larvae tend to be exclusively necrophagous (Heymons and von Lengerken 1931, Peschke et al. 1987, Kočárek 2003).

Even though several aspects of the *O. thoracicum* life histories were described by Heymons and von Lengerken (1931), the authors did not determine a specific relationship between purposefully selected diets and their possible effect on the larval development or survival. In this study, we expanded upon this topic and explored the feeding habits of the *O. thoracicum* larvae. We also aimed to establish the possible influence the different diet sources (animal species) may have on the development time, survival, and body size of the immature stages. Another objective was to determine which of the tested diets is the most appropriate for rearing the species under laboratory conditions.

We believe that the attained results can be valuable for forensic and other experimental research, as they provide robust data on the development of this species which are currently missing. Moreover, this study delivers standardized laboratory techniques for rearing *O. thoracicum* that may be utilized for other related silphid species.

## Materials and Methods

### *O. thoracicum* breeding and experimental setup

Adult beetles were collected from pitfall traps baited with fish (*Pangasianodon hypophthalmus* Sauvage, 1878) and ripened cheese in the forests near Karlštejn town (49°56ʹ14.7″ N 14°11ʹ10.0″ E) in the Czech Republic, during the summer of 2020. Around 100 adults were identified and sexed [using the identification key of Jakubec et al. (2018)] and transferred to the laboratory at the Czech University of Life Sciences Prague, where they were placed inside one large breeding box (Hagen Exo Terra Faunarium large 20 L, dimensions 37 × 22 × 24.5 cm). We followed a methodology comparable to our previous work (Qubaiová et al. 2021). The breeding box contained at least 13 cm of gardening soil, and a small piece of moss, collected from the gathering locality, to sustain humidity and mimic the forest habitat. The substrate was frequently sprinkled with water, and a centrifuge tube (50 ml) filled with water and sealed with cotton, was placed on the surface. The box was kept in a climatic chamber (custom-made by CIRIS s.r.o.) with a constant temperature of 20°C ± 1°C and a photoperiod 16 h day/8 h night (provided by fluorescent light Osram L 8 W/640).

We investigated the effects of diets from three animal sources, cow (*Bos taurus* Linnaeus, 1758), chicken (*Gallus gallus domesticus* Linnaeus, 1758), and the domestic pig (*Sus scrofa* Linnaeus, 1758). The selected tissue parts were beef round muscle (referred to as beef muscle), chicken breast muscle (referred to as chicken muscle), pork back-leg muscle (referred to as pork muscle), and pork liver. The nutritional references for the meats were obtained from the U.S. Department of Agriculture (USDA 2019).

The breeding box was inspected daily for the presence of eggs. Females of *O. thoracicum* lay one egg at a time on the surface or in the shallow part of the soil, a maximum 5 cm below the surface. We collected 331 eggs that were divided equally among the four diet treatments, each was replicated 9 times except for the beef treatment (8 times). Approximately 10 eggs were placed in each Petri dish (10 cm in diameter) filled to three-quarters with moist gardening soil. A piece of specific fresh meat (cc. 5 g.) was placed on the plastic surface in the remaining part of the dish. Subsequently, the Petri dishes were labeled and secured with rubber bands to prevent opening and the possible escape of larvae. Meat was provided ad libitum when the larvae emerged and in all following stages. Additionally, fresh pieces were added if the meat was noticeably consumed, dry, or molded. All Petri dishes were kept under the same conditions as the breeding box and were observed daily for developmental changes and potential molding that was dealt with instantly. The soil inside the dishes was sprinkled with water using a wash bottle when needed.

The development of the following five stages was tracked: first instar larva (L1), second instar larva (L2), third instar larva (L3), postfeeding stage (P.F.), and pupal stage. The egg stage was not included as it is a nonfeeding stage. The change between larval instars was identified by the presence of exuviae. The P.F. stage was identified once the larva formed a clear chamber under the surface.

The emerging L1 larvae were counted and kept together. The transformed L2 larvae were moved to new dishes and kept in groups as well. Upon reaching the third instar phase, all L3 larvae were separated individually in new Petri dishes (10 cm in diameter) and provided with fresh meat ad libitum. The dishes were then placed in an upright position. This separation was helpful for easier identification of the individual larvae and observation of the remaining developmental stages. Also, it gave the larvae sufficient space without competing or colliding with each other when forming the pupation chamber upon reaching the postfeeding stage. The remaining developmental stages were monitored without any significant interference, merely for moistening the soil in the Petri dishes and removing decayed meat when the larvae entered the postfeeding stage. After reaching the adult stage, the beetles were sexed using the identification key of Jakubec et al. (2018).

For obtaining information about the pronotal width, the L3 larvae were temporarily mechanically immobilized; each larva was positioned on a stack of round cotton pads enclosed inside a small dish (6 cm) and gently covered with the dishes’ cover. Photographs were obtained using a Canon macro photo lens MP-E 65 mm on a Canon 550D body. The larvae were then returned to their designated Petri dishes to continue their development. The size data were acquired by measuring the pronotal width from the photographs using the “EidosMicro” program.

### Statistical Analysis

The relationship between the logarithm of the pronotal width, the diet, and the sex of each individual was examined. The identification (I.D.) of the Petri dish, where the larvae were placed originally, was used as a random explanatory variable. We constructed three linear mixed effect models (LMM): Size1, Size2, and Size3. Size1 represented the null model without the explanatory variable sex. Size2 contained all the explanatory variables additively, and Size3 presumed the interaction between sex and the diet. The models were compared using the Bayesian information criterion (BIC). The winning model was examined by the post-hoc analysis (Tukey’s contrasts), with adjusted *p* values by the Benjamini and Hochberg method (B. H. method) (Benjamini and Hochberg 1995) using “glht” function (R package multcomp).

The LMMs were used to explore the relationship between: 1) the total developmental time and the fixed explanatory variable of the diet; 2) the developmental time of each stage and the diet. The Petri dish I.D. was used as a random explanatory variable. The above-mentioned post-hoc analysis was applied to resolve the potential significant difference among the diets.

The survival data were evaluated by the nonparametrical log rank test, which uses the function survdiff from the R package “survival” (Therneau 2021). The effect of diet on the mortality was investigated at four levels (pork liver, pork muscle, beef muscle, and chicken muscle) for all developmental stages except the egg stage. The B.H. method of correction was used for the multiple comparisons.

Performance of the models and deviation from their theoretical assumptions were evaluated by visualizing the corresponding residual plots. The significance level was set at 5% for the p values and 2 points for the Bayesian information criterion. Data management and all analyses were carried out using the R statistical program (R Core Team 2020). Graphical outputs were created using ggplot2 and sjPlot packages (Wickham 2009, Lüdecke 2020).

## Results

In total, 203 larvae completed their development to adulthood (109 females, 93 males, and one individual was deformed thus not sexed), of which 61 individuals were reared on pork liver, 59 on pork muscle, 42 on beef muscle, and 41 on chicken muscle.

### Size Disparity

**Fig. 1.**
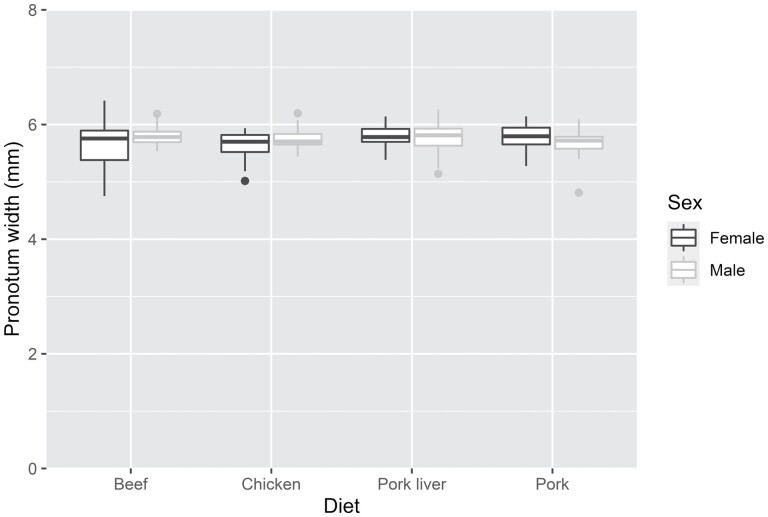
Boxplot graphs of the pronotal width values of *O. thoracicum* L3 larvae reared on different diets for both males and females. Horizontal lines within the boxes indicate median values; upper and lower boxes indicate the 75th and 25th percentiles, respectively. Whiskers indicate the values within the 1.5 interquartile ranges.

Comparison of the models indicated that the simplest one (Size1), with only diet as an explanatory factor, had the lowest values of BIC. Therefore, we did not confirm variations in the pronotal width between the males and females of *O. thoracicum* (see Fig. 1). Furthermore, the pronotal width did not vary among the tested diets (*F* value = 1.73, *p* value = 0.163) (see Fig. 1). The model showed only a close-to significant *p* value between the pronotum width of larvae fed with beef and pork liver diets (*t* value = 1.942, *p* value = 0.0536) though, the post-hoc analysis did not confirm this pattern (*z* value = 1.942, *p* value = 0.187).

### Development Time

**Fig. 2.**
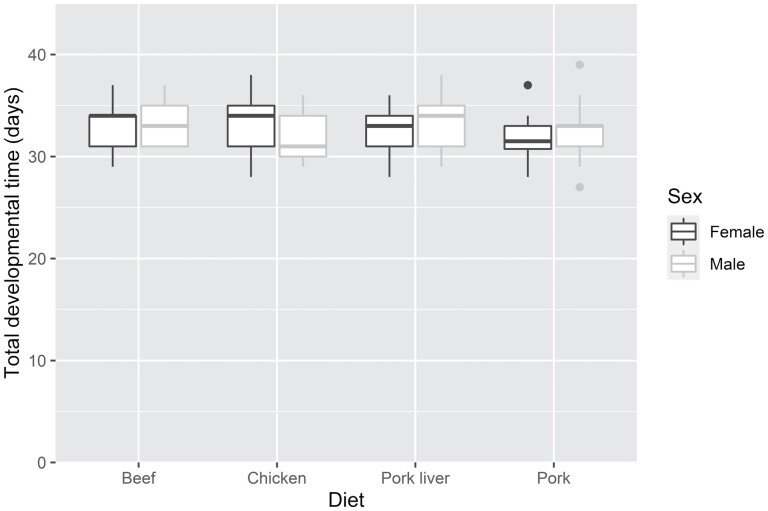
Boxplot graphs of the total development time of *O. thoracicum* males and females reared on different diets from eclosion to adulthood. Horizontal lines within the boxes indicate median values; upper and lower boxes indicate the 75th and 25th percentiles, respectively. Whiskers indicate the values within the 1.5 interquartile ranges.

We did not find any significant effect of the tested diets on the total developmental time (excluding the egg stage). Beetles in all treatments required similar time to develop (beef = 33.143 ± 2.031; chicken = 32.525 ± 2.773; pork liver = 32.901 ± 2.196; pork = 31.9091 ± 2.154 d) (Fig. 2).

**Fig. 3.**
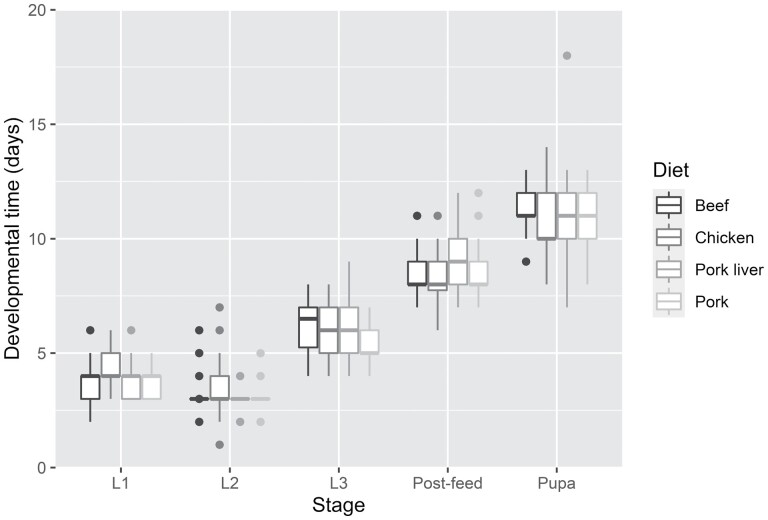
Boxplot graphs of the developmental times of *O. thoracicum* on the various diets at different stages. Horizontal lines within the boxes indicate median values; upper and lower boxes indicate the 75th and 25th percentiles, respectively. Whiskers indicate the values within the 1.5 interquartile ranges.

The more detailed developmental model, which focused on the stage specific developmental times, also did not show any significant influence of the diets in any specific stage (*p* value > 0.05). However, the duration of the first instar was significantly different when compared to other stages (second instar [*t* value = −7.025, *p* value < 0.001]; third instar [*t* value = 21.436, *p* value < 0.001]; postfeeding [*t* value = 48.605, *p* value < 0.001]; pupae [*t* value = 72.831, *p* value < 0.001]) (see Fig. 3).

### Survival

The pairwise comparison that uses the Log-rank test of the survival data revealed a significant difference among four diet combinations: chicken and pork muscle (0.0079), chicken muscle and pork liver (0.0079), beef and pork muscle (0.0079), and beef muscle and pork liver (0.0079). We did not find any significant disparity between beef and chicken muscle nor between pork muscle and pork liver. See Table 1 for the complete summary of the survival model parameters at different stages of development.

**Table 1.** Survival model for *O. thoracicum* throughout six developmental stages

Stage	N. event	Survival	Std. error	Lower 95% CI	Upper 95% CI
**Beef**					
L1	7	0.877	0.0435	0.796	0.967
L2	3	0.825	0.0504	0.732	0.929
L3	2	0.789	0.0540	0.690	0.903
Postfeeding	2	0.754	0.0570	0.651	0.875
Pupa	1	0.737	0.0583	0.631	0.861
**Chicken**					
L1	6	0.893	0.0413	0.815	0.978
L2	5	0.804	0.0531	0.706	0.915
L3	2	0.768	0.0564	0.665	0.887
Postfeeding	1	0.750	0.0579	0.645	0.872
Pupa	1	0.732	0.0592	0.625	0.858
**Pork liver**					
L1	3	0.955	0.0256	0.906	1.000
L2	0	0.955	0.0256	0.906	1.000
L3	0	0.955	0.0256	0.906	1.000
Postfeeding	4	0.924	0.0326	0.863	0.990
Pupa	0	0.924	0.0326	0.863	0.990
**Pork**					
L1	1	0.984	0.0155	0.954	1.000
L2	1	0.969	0.217	0.927	1.000
L3	0	0.969	0.217	0.927	1.000
Postfeeding	2	0.938	0.0303	0.880	0.999
Pupa	1	0.922	0.0335	0.858	0.990

N. event = number of deaths in the group occurring during specified time intervals, Survival = proportion of surviving individuals at the beginning of the stage supplemented with standard errors (Std. error), also lower and upper confidence intervals (Lower 95% CI and Upper 95% CI respectively).

## Discussion

We examined the influence of four diets (chicken, beef, pork muscle, and pork liver) from three animal sources on the development time, survival, and size of the *O. thoracicum* larval stages. Our findings revealed that none of the introduced diets substantially affected the development time. The larvae were able to finish their development on all tested diets with a similar development time, even though there was variability in the nutritional composition of the treatments. This contradicts our previous experiment on the larvae of *Thanatophilus rugosus* (Linnaeus, 1758) (Coleoptera: Silphidae) (Qubaiová et al. 2021), where the diet directly influenced the development time; the fastest development was observed in larvae fed with pork liver closely followed by pork muscle, and the longest development was noted in chicken muscle. The average development time for *O. thoracicum* across all diet treatments we tested was 32.6 d, which was close to Heymons and von Lengerken (1931) study; average of 34.2 d at the mean temperature of 19.25°C, even though their methodology was very simplified. Additionally, the individual stages in all diet treatments were also very similar or even identical in their duration (see Table 2). The 1.6-day disparity between the two studies is minor. It could be caused by the slightly lower temperature in the former author’s work, the variable humidity conditions, data acquirement, or even differences in the feeding and rearing methodology.

**Table 2.** Comparison of original Heymons and von Lengerken (1931) (H & L) developmental data for six individuals at an average 19.25°C, and our data recorded at ±20°C for 198 individuals

Stage	Current study		H & L	
	D	SE	D	SE
L1	3.86	0.05	3.5	0.43
L2	3.18	0.05	3.3	0.33
L3	5.96	0.07	6.3	0.80
Postfeeding	8.62	0.08	10.2	1.40
Pupa	10.98	0.09	10.8	0.70
**Total**	32.6	0.16	34.2	0.31

Developmental time (D) and standard error (SE) are represented in days.

Interestingly, the *O. thoracicum* results were similar to most studies carried on necrophagous dipteran larvae, where the diets tested (whether muscle or internal organ tissues) had no significant influence on the developmental time (e.g., Boatright and Tomberlin 2010, Donato and Liria 2016, Thomas et al. 2016, Bernhardt et al. 2017). However, in a few cases, the diet indeed had an effect (e.g., Clark et al. 2006, Ireland and Turner 2006, Warren and Anderson 2009, Thyssen et al. 2014). Such disparities in the diet influence could be attributed to the probable differences in the applied methodology, or more importantly to the varying nutritional necessities of each necrophagous species, and the general utilization of the food in terms of digestion and processing, which promotes reaching the critical weight during development.

Nonetheless, the diets tested directly affected the survival rates of the *O. thoracicum* larvae, as the highest numbers were obtained when feeding the larvae with both pork liver and muscle. On the other hand, high mortality was noticed with the chicken treatment, in addition to the increased morphological deformations detected in the developing individuals. These results were in accord with the ones obtained from the diet experiments on the *T. rugosus* larvae (Qubaiová et al. 2021). We previously attributed our findings to the lower nutritional quality of chicken muscle when compared to the highly nutritious pork liver or even pork muscle. These conclusions appear equally true for *O. thoracicum* larvae.

The only diet not tested in our previous work was beef muscle, which showed comparable mortality rates to the chicken muscle. Even though, the content of some essential nutritional components vary (information obtained from USDA 2019), e.g., the total fat (5.53 g beef, 2.62 g chicken), iron (2.53 mg beef, 0.37 mg chicken), and cholesterol (61 mg beef, 73 mg chicken) yet, it was not sufficient for achieving different mortality results between the two meats. The beef muscle displayed poorer survival results than both pork liver and muscle, although for instance, the total fat content was almost the same in both beef and pork muscle (5.53 mg, 5.41 mg respectively), and the iron content was higher in the former (2.53 mg, 1.01 mg respectively). Furthermore, there were no substantial differences in the survival rates on either the pork liver or pork muscle. Though, pork liver has a much higher cholesterol content (301 mg pork liver, 68 mg pork muscle) and iron content (23.3 mg pork liver, 1.01 mg pork muscle). Overall, such inconsistencies may be ascribed to the diet’s nutritional composition regarding the amounts of the essential macro- and micro-nutrients, and the correct balance among them. The preceding factors are considered vital aspects that can cause substantial alterations in the insect’s life-history traits (House 1969, Chapman 2012).

Compared to the closely related *T. rugosus*, *O. thoracicum* is considered the generalist feeder of the two species. Perhaps this could be tied to the fact that in nature, *T. rugosus* appears earlier on cadavers; within 1–2 d (Matuszewski and Szafałowicz 2013). As opposed to *O. thoracicum*, which appears in the middle stages of decomposition (usually during the second or even the third week) (Matuszewski et al. 2011). In these later stages, the decomposing body is chemically active, and plenty of metabolic waste products from earlier colonizers and bacteria are present, thereby it no longer sustains the same nutritional qualities as in the earlier stages. For this reason, *O. thoracicum* could be more tolerant to the reduced quality of such a food source and is consequently more adapted to feed on alternative sources as well.

Frątczak-Łagiewska et al. (2020) and Qubaiová et al. (2021) previously recommended that, for forensic purposes, it is important to use high-quality food diets, i.e., diets that enable optimal (shortest) developmental times throughout the larval rearing. This outcome is necessary for obtaining developmental data under controlled conditions, that in turn eliminate the overestimation of the PMI and provide time data sets that are as close as possible to the point of death. Nonetheless, each carrion beetle species is unique in its nutritional requirements for achieving ideal development, growth, and survival. Our results suggest differences even among necrophagous genera belonging to the same family, such as *Oiceoptoma* and *Thanatophilus*, hence establishing specified rearing protocols is entailed for the proper analysis of the insect evidence in forensics.

Many researchers suggest taking into consideration the different positions on the dead body from which the entomological evidence is collected. As it is a nonhomogenous combination of various types of tissues and organs that may influence the life histories of the necrophagous insects in general (Kaneshrajah and Turner 2004, Clark et al. 2006, Day and Wallman 2006, Tarone and Benoit 2019). Since our results indicated that the *O. thoracicum* larvae development time was not affected by any of the tested tissues, one may perhaps postulate that these larvae could be collected from cadavers without apprehension to the potential alteration in the developmental data or subsequent PMI calculations.

Usually, the nutritional quality of a diet can be determined not only by measuring growth rates, weight gain, and development time but also by the survival rates to adulthood (Nation 2015). Consequently, we suggest using a high-quality food diet that ensures the highest survival rates for long-lasting and successful breeding for other laboratory rearing purposes. Such diet may be pork liver and muscle for *O. thoracicum*, as both indicated best results from all the diets tested.

Our findings could be of interest not only for forensic entomology but can possibly shed more light on the ecological competition among species on carrion, as some species are more affected by the food quality than others. This usually gives a competitive advantage to species with a generalist feeding behavior. However, since the generalist *O. thoracicum* appears on carrion later than *T. rugosus* (as mentioned above) and both frequent similar ecological niches, we could argue that *O. thoracicum* does not compete directly with *T. rugosus*. Instead, it can live on what *T. rugosus,* and other faster colonizers leave behind, thus allowing the coexistence of more species on a limited food resource such as carrion.

In closing, we proposed a standardized laboratory rearing method for *O. thoracicum*, and presented first robust report of developmental data, which were thus far lacking. These acquired data are considerable and can be further utilized in constructing thermal summation models for this species.
